# Correction: Binding modes of methyl α-d-glucopyranoside to an artificial receptor in crystalline complexes

**DOI:** 10.1039/d2ra90014a

**Published:** 2022-02-28

**Authors:** Linda Köhler, Conrad Hübler, Wilhelm Seichter, Monika Mazik

**Affiliations:** Institut für Organische Chemie, Technische Universität Bergakademie Freiberg Leipziger Strasse 29 09599 Freiberg Germany monika.mazik@chemie.tu-freiberg.de https://tu-freiberg.de/fakultaet2/orgch +49 3731393170 +49 3731392389

## Abstract

Correction for ‘Binding modes of methyl α-d-glucopyranoside to an artificial receptor in crystalline complexes’ by Linda Köhler *et al.*, *RSC Adv.*, 2021, **11**, 22221–22229. DOI: 10.1039/d1ra03390e.

The authors regret the omission of an acknowledgments section from the original article. The correct acknowledgment is as shown below.

The authors gratefully acknowledge that open access funding was provided by the Publication Fund of the Technische Universität Bergakademie Freiberg.

The Royal Society of Chemistry apologises for these errors and any consequent inconvenience to authors and readers.

## Supplementary Material

